# High preoperative CONUT score is associated with treatment failure and mortality after surgery for acute periprosthetic joint infection: a single-centre retrospective cohort study

**DOI:** 10.5194/jbji-11-613-2026

**Published:** 2026-10-13

**Authors:** Víctor Otero-Naveiro, Blanca Ayuso-García, Silvia Pena-Paz, Ricardo Andrés Rión-Giraldo, María José García-Pais, Alejandro López-Pardo Pardo

**Affiliations:** 1 Department of Orthopaedic Surgery and Traumatology, University Hospital Lucus Augusti, 27003, Lugo, Spain; 2 Department of Infectious Diseases, University Hospital Lucus Augusti, 27003, Lugo, Spain

## Abstract

**Introduction:** Acute periprosthetic joint infection (PJI) is associated with substantial morbidity and mortality. The controlling nutritional status (CONUT) score combines serum albumin, total lymphocyte count, and total cholesterol, and may reflect host vulnerability. This study evaluated whether preoperative CONUT is associated with failure and mortality after surgery for acute PJI.

**Methods:** We performed a single-centre retrospective cohort study of patients surgically treated for acute postoperative or acute haematogenous hip or knee PJI between 1 January 2013 and 30 November 2024. PJI was defined according to EBJIS criteria. CONUT was calculated from preoperative blood tests and dichotomised as low ( < 5; normal to mild) or high ( ≥ 5; moderate to severe). Outcomes were defined using an adapted MSIS-ORT classification as failure, total failure including death within 1 year, and overall mortality.

**Results:** Of 169 screened patients, 59 were excluded for missing CONUT data and 90 were included; 42/90 (46.7 %) had high CONUT. High CONUT was associated with higher failure (35.7 % vs. 10.4 %; p=0.004 ), total failure (57.1 % vs. 12.5 %; p<0.001 ), and overall mortality (61.9 % vs. 22.9 %; p<0.001 ). Adjusted analyses confirmed independent associations with failure (odds ratio (OR) 4.24, 95 % confidence interval (CI) 1.34–13.46; p=0.014 ), total failure (OR 9.57, 95 % CI 2.91–31.50; p<0.001 ), and overall mortality (hazard ratio (HR) 2.73, 95 % CI 1.29–5.74; p=0.008 ).

**Conclusions:** High preoperative CONUT was associated with failure, total failure, and overall mortality after surgery for acute PJI. It may support risk stratification but should not be viewed solely as a nutritional marker, as its components may reflect systemic inflammation, bacteraemia, and infection severity.

## 1 Introduction

Periprosthetic joint infection (PJI) remains one of the most serious complications after hip and knee arthroplasty. Registry-based analyses suggest an infection burden close to 1 % in contemporary arthroplasty practice, although rates may be higher in frailer populations: after hip hemiarthroplasty for femoral neck fracture, a recent systematic review reported a pooled deep infection rate of 2.14 %, rising to 2.64 % at 12 months (Springer et al., 2017; Silas et al., 2024). This burden is likely underestimated because debridement, antibiotics, and implant retention (DAIR) procedures are not consistently captured in national registries (Springer et al., 2017; Lenguerrand et al., 2018). PJI is now a leading cause of failure after total knee arthroplasty and an increasingly important cause of revision after total hip arthroplasty, accounting for 21.6 % of knee revisions and 18.2 % of hip revisions in a recent registry-based analysis (Koh et al., 2017; Hauer et al., 2024). Its absolute impact is expected to increase as arthroplasty volumes expand and treated populations become older and more comorbid (Kurtz et al., 2007).

Patients with PJI may require repeated procedures, prolonged antimicrobial treatment, and extended hospitalisation, and may experience persistent functional impairment. Survival is also adversely affected; early PJI after primary total knee arthroplasty has been associated with higher mortality than non-infected controls, reaching 15.7 % at 5 years and 38 % at 10 years (Thompson et al., 2022). More than 20 000 PJIs arising from primary arthroplasties performed in Europe in 2019 were estimated to account for EUR 346 million in reimbursement payments, and septic revision may cost up to three times more than aseptic revision (Alt et al., 2025).

Identifying host-related factors associated with poor outcome is therefore clinically relevant. Malnutrition and impaired immunonutritional status are increasingly recognised as potentially modifiable risk factors in arthroplasty, although reported prevalence varies widely according to the definition used (Cross et al., 2014; Almeida et al., 2024). A recent meta-analysis showed that malnutrition was associated with higher odds of surgical site infection and PJI after total joint arthroplasty (Chen and Chen, 2024). In established PJI, conventional nutritional biomarkers may also provide prognostic information; hypoalbuminaemia is common and has been associated with failure after both one-stage and two-stage revision (Green et al., 2023; Traverso et al., 2023; Li et al., 2024).

The controlling nutritional status (CONUT) score combines serum albumin, total lymphocyte count, and total cholesterol, and was originally developed as a hospital-based nutritional screening tool (de Ulíbarri et al., 2005). These three components are intended to reflect protein reserve, immune-nutritional status, and caloric depletion, respectively. In primary hip and knee arthroplasty, Nutritional Risk Screening 2002 (NRS-2002) and CONUT have been linked to postoperative infectious complications and quality-of-life domains, while admission CONUT has also been associated with 1-year PJI risk after total joint arthroplasty (De Mauro et al., 2026; Guo et al., 2026). In established PJI, moderate-to-severe CONUT has been associated with failure after two-stage exchange (Li et al., 2022). However, the prognostic value of CONUT as a composite host-status marker in acute PJI remains insufficiently defined.

The aim of this study was to determine the prevalence of high CONUT in patients surgically treated for acute hip or knee PJI and to evaluate its association with treatment failure and mortality alongside conventional inflammatory biomarkers and established host-assessment tools.

## 2 Methods

### 2.1 Study design and setting

A retrospective cohort study was conducted using the institutional database of the Bone and Joint Infection Unit. All consecutive adult patients surgically treated for acute hip or knee PJI at a tertiary referral centre between 1 January 2013 and 30 November 2024 were screened for eligibility.

### 2.2 Patient selection

Acute postoperative PJI was defined as infection occurring within 3 months of the index arthroplasty with symptom duration of ≤ 3 weeks. Acute haematogenous PJI was defined as infection with symptom duration of ≤ 3 weeks in a previously well-functioning prosthesis. PJI was defined according to EBJIS criteria; cases fulfilling the “infection confirmed” or “infection likely” categories were eligible (McNally et al., 2021). Patients were excluded if they had non-acute infection, no surgical treatment, simultaneous infection of more than one prosthesis, follow-up < 12 months among survivors, or missing laboratory data required for CONUT calculation.

### 2.3 Surgical management

Surgical strategy reflected routine multidisciplinary practice based on host, implant, soft-tissue, microbiological, and feasibility factors. Procedures were retrospectively classified according to contemporary concepts for acute PJI management (Sigmund et al., 2025). For descriptive analyses, procedures were grouped as implant retention, including DAIR with or without modular component exchange, or implant exchange/removal, including one-stage exchange, two-stage exchange, and resection arthroplasty. Antimicrobial treatment was individualised according to culture results and infectious diseases assessment.

### 2.4 Variables and CONUT score

Baseline variables included age, sex, relevant comorbidities, index arthroplasty indication, McPherson host grade, Joint-Specific BACH classification (JS-BACH), microbiology, blood cultures, inflammatory biomarkers, and CONUT components. McPherson host grade was dichotomised as A–B versus C, and JS-BACH as low (Uncomplicated) versus high (Complex or Limited Options) (Hotchen et al., 2021). CONUT was calculated using the original scoring system described by de Ulíbarri et al. (2005), based on the preoperative values closest to surgery for serum albumin (0–6 points), total lymphocyte count (0–3 points), and total cholesterol (0–3 points). The resulting total score ranges from 0 to 12 and is categorised as normal (0–1), mild (2–4), moderate (5–8), or severe (9–12). Detailed component cut-points are shown in Table S1 in the Supplement. Patients were dichotomised a priori as low CONUT ( < 5) or high CONUT ( ≥ 5), consistent with the original moderate-to-severe CONUT threshold and previous PJI outcome literature (Li et al., 2022). Total cholesterol was retrieved from routine admission or preoperative blood tests and analysed irrespective of fasting status, in line with European guidance supporting non-fasting lipid profiles for routine clinical assessment (Nordestgaard et al., 2016). Difficult-to-treat pathogens were defined using criteria adapted from Wimmer et al. (2020), including fungi and excluding *Cutibacterium acnes* (Table S3). C-reactive protein (CRP), CRP-to-albumin ratio (CAR), and neutrophil-to-lymphocyte ratio (NLR) were analysed as exploratory inflammatory biomarkers (Table S5).

### 2.5 Outcomes

Outcomes were assessed using the Musculoskeletal Infection Society Outcome Reporting Tool (MSIS-ORT), adapted according to Borsinger et al. (2021). Failure comprised tiers 3B–3F: septic revision or reoperation, early aseptic revision, amputation, resection arthroplasty, arthrodesis, or retained spacer. Total failure comprised failure plus death within 1 year from PJI treatment initiation, corresponding to tiers 3B–3F plus 4A. Overall mortality was defined as death from any cause and was analysed as the time from surgery for acute PJI to death or last follow-up. MSIS-ORT categories were mutually exclusive, whereas overall mortality included all deaths from any cause irrespective of the assigned tier.

### 2.6 Statistical analysis

Continuous variables were assessed for normality and are presented as mean ± standard deviation or median with interquartile range. Categorical variables are reported as frequencies and percentages. Comparisons between groups were performed using the student's t test or Mann–Whitney U test for continuous variables, and the $\chi^{2}$ test or Fisher's exact test for categorical variables, as appropriate.

Multivariable logistic regression was used to identify factors independently associated with failure and total failure. Failure-free survival, total failure-free survival, and overall survival were estimated using Kaplan–Meier curves and compared between CONUT groups using the log-rank test. Cox proportional hazards regression was used to analyse overall mortality. Covariates were selected according to clinical relevance and the limited number of events. A sensitivity Cox model analysed CONUT as a continuous variable. Results are reported as odds ratios (ORs) or hazard ratios (HRs) with 95 % confidence intervals (CIs). Statistical analyses were performed using IBM SPSS Statistics for Windows, version 22.0 (IBM Corp., Armonk, NY, USA).

## 3 Results

### 3.1 Baseline characteristics according to CONUT status

A total of 169 patients were screened, of whom 90 were included (86 confirmed infection and 4 infection likely according to EBJIS), after applying the eligibility criteria and excluding cases with missing data required for CONUT calculation (Fig. 1). The cohort comprised 16 hip hemiarthroplasties (17.8 %), 39 total hip arthroplasties (43.3 %), and 35 total knee arthroplasties (38.9 %). Median follow-up was 46.5 months (range, 0–149) overall and 59 months (range, 12–149) among survivors.

**Figure 1:**
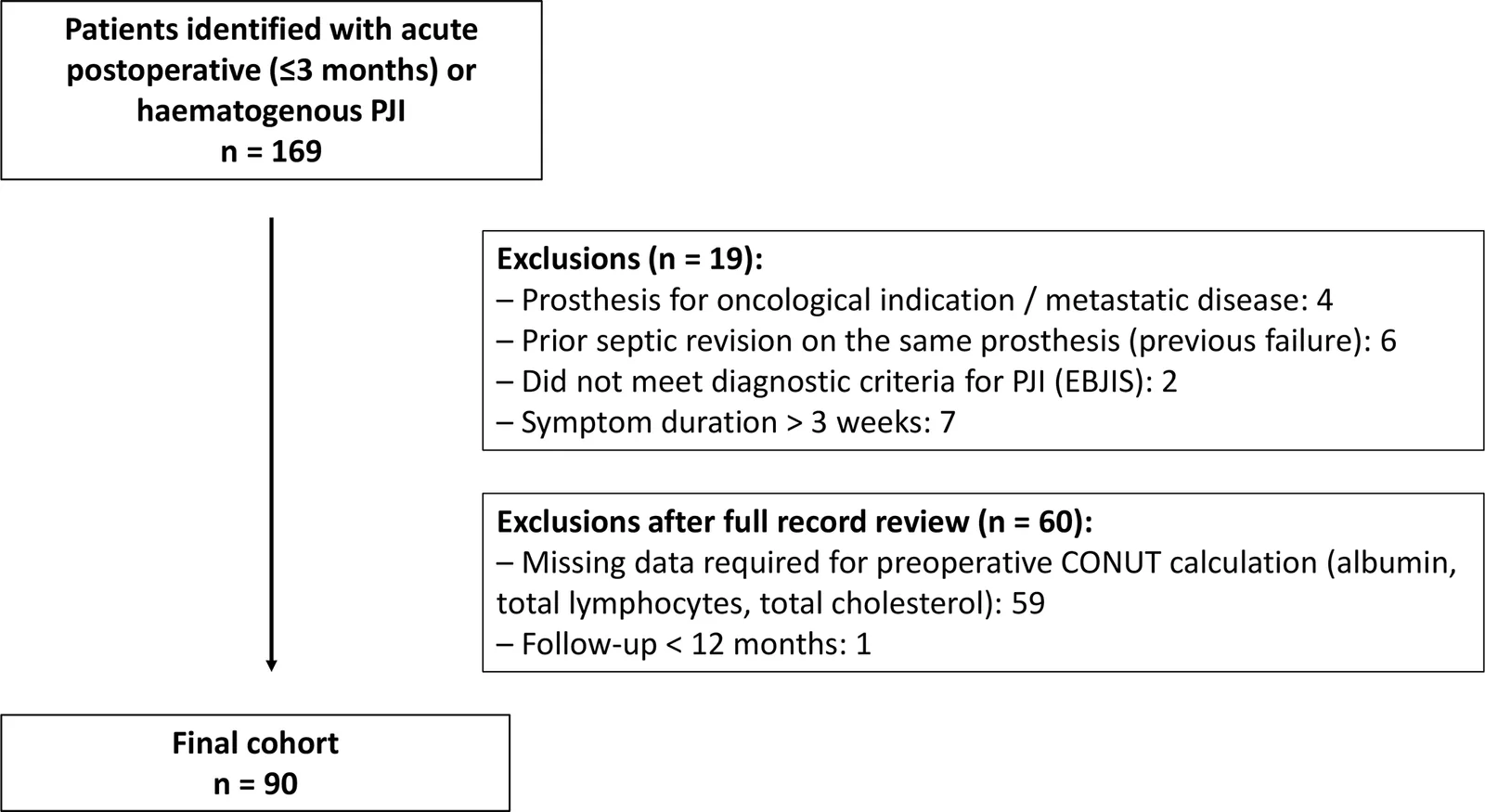
Study flowchart. Patient selection and final cohort.

Laboratory measurements were obtained a mean of 3.3 d before surgery (median 2 d, range 0–15). Forty-two patients (46.7 %) were classified as high CONUT and 48 (53.3 %) as low CONUT. High-CONUT patients were older and more frequently aged ≥ 80 years and McPherson host grade C; preoperative CRP was higher in the high-CONUT group. *Staphylococcus aureus* infection and positive blood cultures were more frequent in the high-CONUT group, while coagulase-negative staphylococci were more common in the low-CONUT group (Tables 1 and S3). In the overall cohort, *S. aureus* infection was associated with positive blood cultures ( 15/33 [45.5 %] vs. 8/57 [14.0 %]; p=0.001 ).

Fifty-nine patients were excluded because at least one CONUT component was unavailable. Included and excluded patients showed no significant baseline differences; high JS-BACH was numerically more frequent among included patients (74.4 % vs. 61.0 %), without reaching statistical significance ( p=0.083 ) (Table S2).

**Table 1:** Baseline characteristics according to CONUT group.

Variable	Overall cohort n=90	High CONUT n=42	Low CONUT n=48	p value
Age, years (mean ± SD)	76.6 ± 10.6	80.0 ± 9.4	73.6 ± 10.8	**0.004**
Age ≥ 80 years, n (%)	35 (38.9)	22 (52.4)	13 (27.1)	**0.014**
Female sex, n (%)	58 (64.4)	28 (66.7)	30 (62.5)	0.680
Diabetes mellitus, n (%)	23 (25.6)	14 (33.3)	9 (18.8)	0.114
Dementia, n (%)	15 (16.7)	10 (23.8)	5 (10.4)	0.089
Malignancy, n (%)	21 (23.3)	13 (31.0)	8 (16.7)	0.110
Fracture/revision indication, n (%)	37 (41.1)	20 (47.6)	17 (35.4)	0.240
Acute haematogenous infection, n (%)	22 (24.4)	13 (31.0)	9 (18.8)	0.179
McPherson host grade C, n (%)	27 (30.0)	19 (45.2)	8 (16.7)	**0.003**
High JS-BACH, n (%)	67 (74.4)	34 (81.0)	33 (68.8)	0.185
CRP, mg L^−1^ (median [IQR])	50.5 [17.5–145.1]	80.5 [36.7–173.0]	38.1 [12.6–110.9]	**0.016**
Polymicrobial infection, n (%)	20 (22.2)	7 (16.7)	13 (27.1)	0.236
*Staphylococcus aureus*, n (%)	33 (36.7)	20 (47.6)	13 (27.1)	**0.044**
Coagulase-negative staphylococci (CoNS), n (%)	19 (21.1)	3 (7.1)	16 (33.3)	**0.002**
Positive blood cultures, n (%)	23 (25.6)	17 (40.5)	6 (12.5)	**0.002**

Bold indicates statistically significant differences ( p<0.05 ). CONUT: controlling nutritional status; SD: standard deviation; IQR: interquartile range; CRP: C-reactive protein; CoNS: coagulase-negative staphylococci; JS-BACH: Joint-Specific BACH classification.

### 3.2 Surgical management and treatment results

Treatment strategies were DAIR with modular parts exchange (DAIR-WMPE) in 40/90 patients (44.4 %), DAIR without modular exchange (DAIR-WO) in 11/90 (12.2 %), one-stage exchange in 14/90 (15.6 %), two-stage exchange in 21/90 (23.3 %), and resection arthroplasty in 4/90 (4.4 %). Overall, 51/90 patients (56.7 %) underwent implant retention and 39/90 (43.3 %) underwent implant exchange/removal; implant retention was not associated with failure, total failure, or overall mortality (Table 2).

Total failure rates were 10/40 (25.0 %) after DAIR-WMPE, 6/11 (54.5 %) after DAIR-WO, 4/14 (28.6 %) after one-stage exchange, 6/21 (28.6 %) after two-stage exchange, and 4/4 (100 %) after resection arthroplasty. These treatment-specific rates were considered descriptive because of small subgroups and confounding by indication.

### 3.3 Outcome distribution and univariate associations

According to MSIS-ORT, Tier 1 was achieved in 42/90 patients (46.7 %) at final follow-up (median, 46.5 months; range, 0–149). No patients were classified as Tier 2 or Tier 3F. Failure occurred in 20 patients (22.2 %), total failure in 30 (33.3 %), and overall mortality in 37 (41.1 %). High CONUT was associated with higher failure (35.7 % vs. 10.4 %; p=0.004 ), total failure (57.1 % vs. 12.5 %; p<0.001 ), and overall mortality (61.9 % vs. 22.9 %; p<0.001 ) (Fig. 2; Table S4).

**Figure 2:**
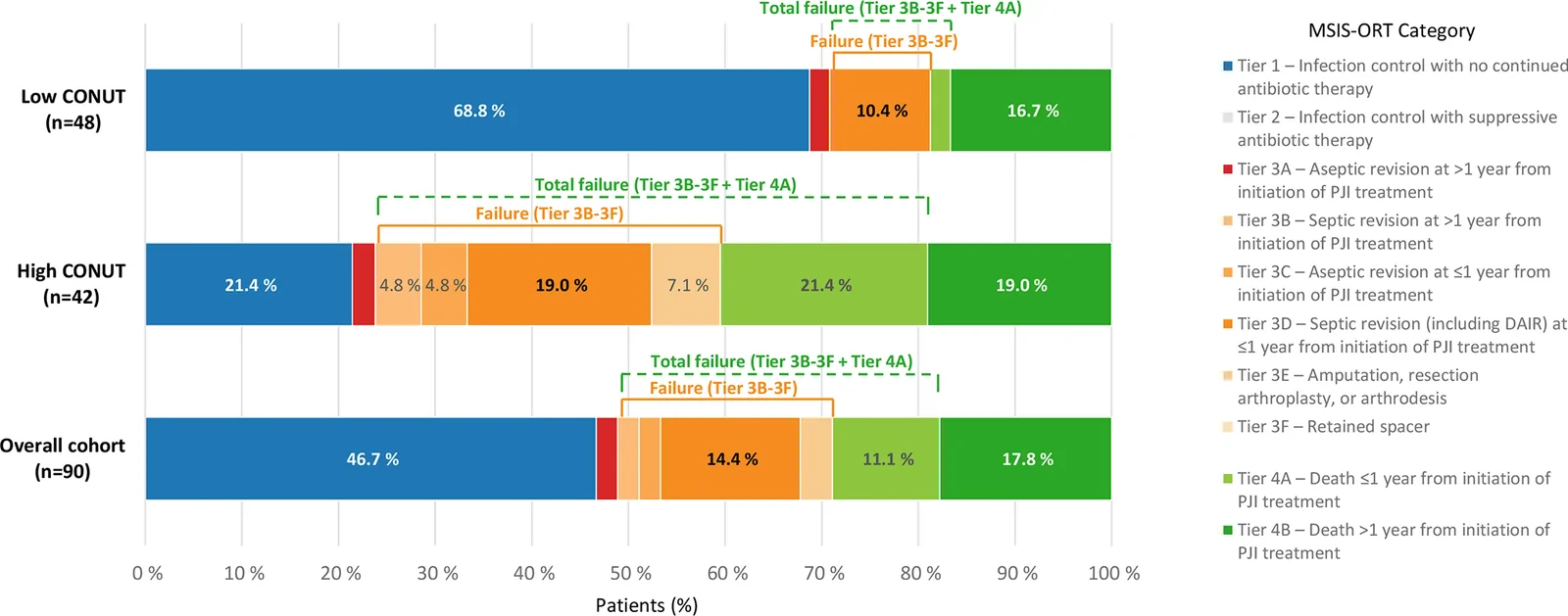
MSIS-ORT distribution according to CONUT status. Stacked bar chart showing mutually exclusive MSIS-ORT categories according to preoperative CONUT group. MSIS-ORT: Musculoskeletal Infection Society Outcome Reporting Tool; CONUT: controlling nutritional status; PJI: periprosthetic joint infection; DAIR: debridement, antibiotics, and implant retention.

In univariate analyses, high CONUT showed the most consistent association across outcomes (Table 2). Diabetes mellitus was associated with all three outcomes; McPherson host grade C, high JS-BACH, malignancy, and fracture/revision indication with total failure; and age with overall mortality. CRP, CAR, and NLR showed no consistent association across outcomes (Table S5).

**Table 2:** Main univariate associations with the study outcomes. For categorical variables, data are presented as events/total (%) for the category vs. the reference group; continuous data are mean ± SD or median [IQR] for patients with vs. without the outcome.

Variable	Failure	p value	Total failure	p value	Overall mortality	p value
Age, years (mean ± SD)	74.7 ± 9.3 vs. 77.1 ± 11.0	0.372	78.3 ± 10.3 vs. 75.7 ± 10.8	0.279	82.4 ± 8.9 vs. 72.5 ± 9.9	<0.001
Age ≥ 80 years, n (%)	7/35 (20.0) vs. 13/55 (23.6)	0.686	15/35 (42.9) vs. 15/55 (27.3)	0.126	24/35 (68.6) vs. 13/55 (23.6)	<0.001
Female sex, n (%)	13/58 (22.4) vs. 7/32 (21.9)	0.953	19/58 (32.8) vs. 11/32 (34.4)	0.876	23/58 (39.7) vs. 14/32 (43.8)	0.705
Diabetes mellitus, n (%)	10/23 (43.5) vs. 10/67 (14.9)	**0.004**	15/23 (65.2) vs. 15/67 (22.4)	<0.001	14/23 (60.9) vs. 23/67 (34.3)	**0.026**
Dementia, n (%)	5/15 (33.3) vs. 15/75 (20.0)	0.309	8/15 (53.3) vs. 22/75 (29.3)	0.072	10/15 (66.7) vs. 27/75 (36.0)	**0.028**
Malignancy, n (%)	8/21 (38.1) vs. 12/69 (17.4)	0.070	12/21 (57.1) vs. 18/69 (26.1)	**0.008**	11/21 (52.4) vs. 26/69 (37.7)	0.231
High CONUT, n (%)	15/42 (35.7) vs. 5/48 (10.4)	**0.004**	24/42 (57.1) vs. 6/48 (12.5)	<0.001	26/42 (61.9) vs. 11/48 (22.9)	<0.001
McPherson host grade C, n (%)	9/27 (33.3) vs. 11/63 (17.5)	0.097	16/27 (59.3) vs. 14/63 (22.2)	**0.001**	14/27 (51.9) vs. 23/63 (36.5)	0.175
High JS-BACH, n (%)	18/67 (26.9) vs. 2/23 (8.7)	0.071	27/67 (40.3) vs. 3/23 (13.0)	**0.017**	30/67 (44.8) vs. 7/23 (30.4)	0.228
Fracture/revision indication, n (%)	8/37 (21.6) vs. 12/53 (22.6)	0.909	17/37 (45.9) vs. 13/53 (24.5)	**0.034**	20/37 (54.1) vs. 17/53 (32.1)	**0.037**
Acute haematogenous infection, n (%)	8/22 (36.4) vs. 12/68 (17.6)	0.081	9/22 (40.9) vs. 21/68 (30.9)	0.386	9/22 (40.9) vs. 28/68 (41.2)	0.982
Implant retention, n (%)	10/51 (19.6) vs. 10/39 (25.6)	0.495	16/51 (31.4) vs. 14/39 (35.9)	0.652	22/51 (43.1) vs. 15/39 (38.5)	0.655
Albumin, g dL^−1^ (mean ± SD)	3.17 ± 0.28 vs. 3.35 ± 0.52	**0.047**	3.04 ± 0.37 vs. 3.45 ± 0.48	<0.001	3.05 ± 0.37 vs. 3.50 ± 0.47	<0.001
Total lymphocyte count, cells µ L^−1^ (median [IQR])	1100 [725–1650] vs. 1500 [1000–2025]	**0.021**	1150 [775–1700] vs. 1500 [1100–2100]	**0.011**	1200 [900–1650] vs. 1600 [1055–2100]	**0.032**
Total cholesterol, mg dL^−1^ (median [IQR])	128.5 [113.5–151.3] vs. 149.0 [118.0–171.0]	0.151	128.5 [110.0–147.8] vs. 153.0 [122.8–173.5]	**0.006**	136.0 [112.0–166.5] vs. 147.0 [124.0–170.5]	0.226
*Staphylococcus aureus*, n (%)	10/33 (30.3) vs. 10/57 (17.5)	0.161	13/33 (39.4) vs. 17/57 (29.8)	0.353	13/33 (39.4) vs. 24/57 (42.1)	0.801
Coagulase-negative staphylococci (CoNS), n (%)	1/19 (5.3) vs. 19/71 (26.8)	0.061	3/19 (15.8) vs. 27/71 (38.0)	0.068	4/19 (21.1) vs. 33/71 (46.5)	**0.045**
Positive blood cultures, n (%)	9/23 (39.1) vs. 11/67 (16.4)	**0.024**	12/23 (52.2) vs. 18/67 (26.9)	**0.026**	9/23 (39.1) vs. 28/67 (41.8)	0.823

Bold indicates statistically significant differences ( p<0.05 ). CONUT: controlling nutritional status; SD: standard deviation; IQR: interquartile range; JS-BACH: Joint-Specific BACH classification; CoNS: coagulase-negative staphylococci.

### 3.4 Multivariable analysis

In multivariable logistic regression, high CONUT and diabetes mellitus remained independently associated with failure and total failure. For failure, the adjusted ORs were 4.24 (95 % CI 1.34–13.46; p=0.014 ) for high CONUT and 3.81 (95 % CI 1.25–11.59; p=0.019 ) for diabetes mellitus. For total failure, high CONUT (OR 9.57, 95 % CI 2.91–31.50; p<0.001 ), diabetes mellitus (OR 9.62, 95 % CI 2.56–36.20; p=0.001 ), and fracture/revision indication (OR 3.78, 95 % CI 1.17–12.27; p=0.027 ) remained independently associated with the outcome (Table 3).

Kaplan–Meier analyses showed significantly lower failure-free survival in the high-CONUT group (15/42 vs. 5/48 events; log-rank $\chi^{2}=11.140$ ; p=0.001 ), lower total-failure-free survival (24/42 vs. 6/48 events; log-rank $\chi^{2}=20.623$ ; p<0.001 ), and lower overall survival (26/42 vs. 11/48 deaths from any cause; log-rank $\chi^{2}=16.233$ ; p<0.001 ). Median overall survival was 42.3 months (95 % CI 27.1–57.4) in the high-CONUT group and was not reached in the low-CONUT group.

In the Cox model for overall mortality, older age (HR 1.13 per year, 95 % CI 1.07–1.19; p<0.001 ), diabetes mellitus (HR 3.43, 95 % CI 1.63–7.22; p=0.001 ), fracture/revision indication (HR 2.26, 95 % CI 1.09–4.69; p=0.029 ), and high CONUT (HR 2.73, 95 % CI 1.29–5.74; p=0.008 ) were independently associated with overall mortality (Fig. 3; Table 3). In an exploratory sensitivity Cox model replacing high CONUT with continuous CONUT, each 1-point increase in CONUT remained associated with higher overall mortality (HR 1.34, 95 % CI 1.14–1.57; p<0.001 ), together with older age (HR 1.13 per year, 95 % CI 1.07–1.19; p<0.001 ), diabetes mellitus (HR 4.25, 95 % CI 2.01–9.01; p<0.001 ), and fracture/revision indication (HR 2.18, 95 % CI 1.04–4.55; p=0.039 ).

**Figure 3:**
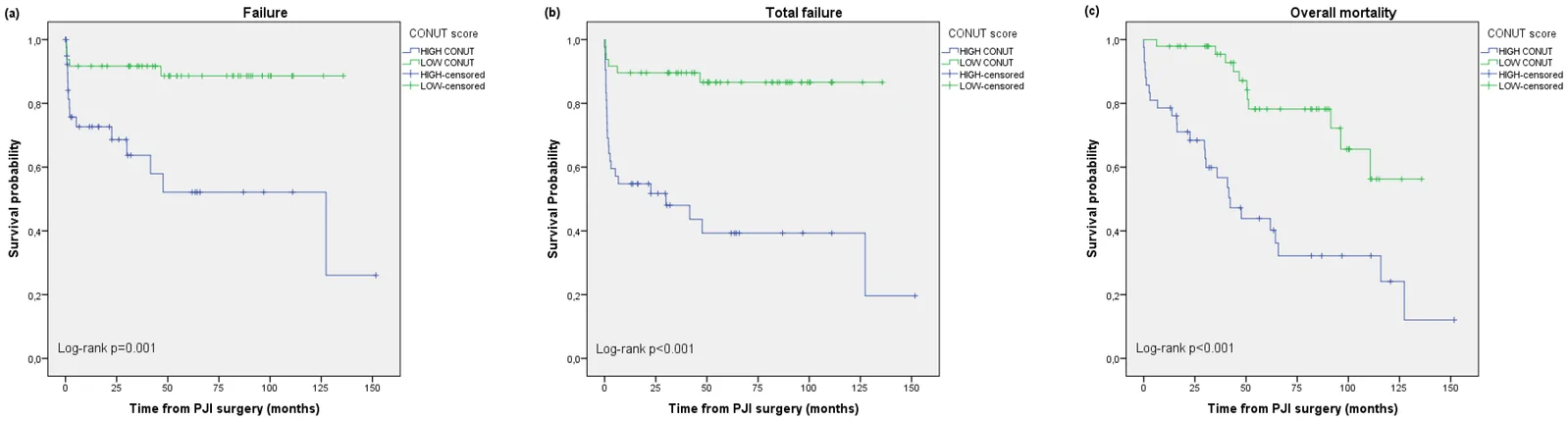
Kaplan–Meier curves for outcomes according to preoperative CONUT status. Kaplan–Meier curves showing **(a)** failure-free survival, **(b)** total failure-free survival, and **(c)** overall survival, after surgery for acute PJI. Tick marks indicate censored observations. PJI: periprosthetic joint infection; CONUT: Controlling Nutritional Status.

**Table 3:** Final multivariable models.

Outcome	Variable	Effect size (95 % CI)	p value
Failure	High CONUT	OR 4.24 (1.34–13.46)	**0.014**
	Diabetes mellitus	OR 3.81 (1.25–11.59)	**0.019**
Total failure	High CONUT	OR 9.57 (2.91–31.50)	<0.001
	Diabetes mellitus	OR 9.62 (2.56–36.20)	**0.001**
	Fracture/revision indication	OR 3.78 (1.17–12.27)	**0.027**
Overall mortality	Age (per year)	HR 1.13 (1.07–1.19)	<0.001
	Diabetes mellitus	HR 3.43 (1.63–7.22)	**0.001**
	Fracture/revision indication	HR 2.26 (1.09–4.69)	**0.029**
	High CONUT	HR 2.73 (1.29–5.74)	**0.008**

Bold indicates statistically significant associations ( p<0.05 ). OR: odds ratio; HR: hazard ratio; CI: confidence interval; CONUT: controlling nutritional status.

## 4 Discussion

High preoperative CONUT identified patients with worse crude and time-to-event outcomes after surgery for acute PJI and remained independently associated with failure, total failure, and overall mortality after adjustment. The sensitivity analysis using continuous CONUT showed a similar association with overall mortality, consistent with previous evidence of increasing PJI-related risk at higher scores (Li et al., 2022; Guo et al., 2026). These findings support CONUT as a marker of preoperative host vulnerability, although the limited number of events warrants cautious interpretation.

Previous studies similarly linked hypoalbuminaemia with poor outcomes in established PJI. After one-stage septic revision, Traverso et al. (2023) found hypoalbuminaemia to be the only independent predictor of failure (OR 5.64), whereas total lymphocyte count and their combined malnutrition definition were not associated with outcome. Green et al. (2023) reported higher failure after first-stage resection in patients with albumin < 3.5 g dL^−1^ (adjusted OR 2.50), and Li et al. (2024) found that hypoalbuminaemia at both the first stage and reimplantation was associated with failure, with worse outcomes when it persisted until reimplantation.

In our cohort, CRP, CAR, and NLR did not show consistent associations across outcomes; Hong et al. (2019) similarly found no association between preoperative CAR and failure after two-stage exchange. Although this does not diminish their diagnostic or monitoring value, isolated inflammatory indices may provide a less consistent prognostic signal than composite host-response measures (Ali et al., 2024). High-CONUT patients more frequently had *Staphylococcus aureus* infection and positive blood cultures, whereas coagulase-negative staphylococci were more common in the low-CONUT group. The association between *S. aureus* and positive blood cultures was consistent with a more systemic infection phenotype. This accords with Campbell et al. (2022), who linked low albumin with culture-confirmed *S. aureus* musculoskeletal infection; and Li et al. (2024), who associated hypoalbuminaemia in chronic PJI with potentially more virulent organisms, including *S. aureus*. Hypoalbuminaemia and high CONUT may therefore reflect both host vulnerability and the systemic response to a more invasive infection phenotype, although this interpretation remains exploratory and does not establish causality.

Importantly, CONUT should not be interpreted as a formal diagnosis of malnutrition, particularly in acute PJI. According to the Global Leadership Initiative on Malnutrition (GLIM) framework, malnutrition requires at least one phenotypic and one aetiological criterion, rather than serum-based abnormalities alone (Cederholm et al., 2019). The updated European Society for Clinical Nutrition and Metabolism (ESPEN) guidance makes a similar distinction: albumin is prognostic and may identify patients at increased metabolic risk, but it should not be considered a nutritional marker per se (Weimann et al., 2025).

This distinction is especially relevant in our cohort because CONUT components were measured after symptom onset and close to surgery. In acute infection, systemic inflammation, bacteraemia, metabolic stress, and catabolic activation may impair the host's ability to maintain normal circulating albumin, lymphocyte, and cholesterol levels. Hypoalbuminaemia may reflect the hepatic acute-phase response and reduced albumin synthesis during systemic inflammation (Beyer et al., 2022). Lymphopenia may accompany immune dysregulation and bacteraemia, and total lymphocyte count may correlate poorly with nutritional status in older adults (de Jager et al., 2010; Kuzuya et al., 2005; Wang et al., 2024). Hypocholesterolaemia has also been linked to sepsis severity and mortality, probably reflecting the interaction between inflammation, lipid metabolism, and host response (Taylor et al., 2024). In acute PJI, CONUT may therefore be better understood as a composite host-response marker integrating nutritional and physiological reserve, inflammation, immune dysfunction, and catabolic stress. Without pre-infection measurements, however, we cannot distinguish pre-existing vulnerability from infection-induced deterioration.

Diabetes mellitus was independently associated with all three outcomes, consistent with DAIR literature identifying diabetes among relevant comorbidities (Abbaszadeh et al., 2026). Lin et al. (2022) reported higher reinfection after two-stage revision with HbA1c ≥ 7 %, although no definitive threshold was established. This association is biologically plausible because hyperglycaemia impairs neutrophil chemotaxis, phagocytosis, and bactericidal activity (Jafar et al., 2016). Mortality was also associated with age and fracture/revision indication, indicating that CONUT should complement rather than replace established PJI-specific host and complexity scores such as JS-BACH by adding an objective laboratory-based dimension of host status.

Strengths included the use of EBJIS diagnostic criteria and structured MSIS-ORT outcome assessment. This is relevant because a lack of standardisation in PJI outcome reporting remains a major limitation, and different definitions can substantially alter reported success rates (Borsinger et al., 2021; Tarabichi et al., 2025). The MSIS-ORT allowed transparent reporting of mutually exclusive outcome categories and helped to distinguish failure, total failure, and overall mortality.

The main limitation of this study was the exclusion of otherwise eligible patients because one or more laboratory components required to calculate CONUT were unavailable, which may have introduced selection bias. In our centre, albumin and total cholesterol are usually included in standard morning admission or preoperative laboratory profiles but are not consistently requested in urgent emergency department blood tests. This probably explains why CONUT components were missing in a substantial proportion of patients. However, included patients and those excluded because of missing CONUT components showed no significant differences in baseline demographic, clinical, host-related, inflammatory, microbiological, or blood culture characteristics, mitigating but not eliminating this risk. Other limitations include the retrospective single-centre design, modest sample size, limited number of events, and residual confounding. Surgical treatment was heterogeneous and non-randomised, reflecting routine clinical decision-making; therefore, treatment-specific comparisons should be considered descriptive rather than causal.

## 5 Conclusions

High preoperative CONUT was associated with failure, total failure, and overall mortality after surgery for acute hip and knee PJI. As a simple and widely available composite host-status marker, CONUT may support preoperative risk stratification in this high-risk population. However, in acute PJI, CONUT should not be interpreted solely as a nutritional marker because its components may be influenced by systemic inflammation, bacteraemia, and infection severity. Larger multi-centre studies should validate these findings and determine whether targeted optimisation strategies can modify these outcomes.

## Supplement

10.5194/jbji-11-613-2026-supplement The supplement related to this article is available online at https://doi.org/10.5194/jbji-11-613-2026-supplement.

## Data Availability

No custom code was developed for this study. The patient-level dataset is not publicly available because it derives from retrospective medical records and contains sensitive clinical information. Public deposition is not covered by the study's ethical approval and is restricted by confidentiality obligations, institutional policies, and applicable data-protection legislation. Access to an appropriately anonymised and limited dataset may be considered upon reasonable request, subject to prior institutional and research ethics approval.
