# Supplementary material for: High preoperative CONUT score is associated with treatment failure and mortality after surgery for acute periprosthetic joint infection: a single-centre retrospective cohort study
Source: J Bone Jt Infect. 2026 Oct 13;11(5):613–22. doi: 10.5194/jbji-11-613-2026 (PMC13662809; doi:10.5194/jbji-11-613-2026)
Supplement: Supplementary file 1 [file jbji-11-613-2026-supplement.pdf]

Supplement of J. Bone Joint Infect., 11, 613–622, 2026
https://doi.org/10.5194/jbji-11-613-2026-supplement
© Author(s) 2026. CC BY 4.0 License.

Supplement of

High preoperative CONUT score is associated with treatment failure and mortality after surgery for acute periprosthetic joint infection: a single-centre retrospective cohort study

Víctor Otero-Naveiro et al.

Correspondence to: Víctor Otero-Naveiro (correodevictorotero@hotmail.com)

The copyright of individual parts of the supplement might differ from the article licence.

Supplementary Table S1. Calculation and interpretation of the CONUT score

Parameter	Normal	Mild	Moderate	Severe
**Serum albumin, g/dL**	≥3.5<br>0 points	3.0–3.49<br>2 points	2.5–2.99<br>4 points	<2.5<br>6 points
**Total lymphocyte count, cells/μL**	≥1600<br>0 points	1200–1599<br>1 point	800–1199<br>2 points	<800<br>3 points
**Total cholesterol, mg/dL**	>180<br>0 points	140–180<br>1 point	100–139<br>2 points	<100<br>3 points
**Total CONUT score**	**0–1**	**2–4**	**5–8**	**9–12**

The total CONUT score is obtained by adding the component scores. In the present study, scores <5 were classified as low CONUT (normal-to-mild) and scores ≥5 as high CONUT (moderate-to-severe). CONUT: Controlling Nutritional Status.

Supplementary Table S2. Comparison between included patients and patients excluded because of missing CONUT components

Variable	Included patients n=90	Excluded patients n=59	p value
Age, years (mean ± SD)	76.6 ± 10.6	73.8 ± 9.9	0.117
Age ≥ 80 years, n (%)	35 (38.9)	16 (27.1)	0.139
Female sex, n (%)	58 (64.4)	31 (52.5)	0.147
Diabetes mellitus, n (%)	23 (25.6)	14 (23.7)	0.801
Dementia, n (%)	15 (16.7)	7 (11.9)	0.419
Malignancy, n (%)	21 (23.3)	8 (13.6)	0.141
Fracture/revision indication, n (%)	37 (41.1)	19 (32.2)	0.272
Acute haematogenous infection, n (%)	22 (24.4)	19 (32.2)	0.300

McPherson host grade C, n (%)	27 (30.0)	13 (22.0)	0.283
High JS-BACH, n (%)	67 (74.4)	36 (61.0)	0.083
CRP, mg/L (median [IQR])	50.5 [17.5–145.1]	62.7 [16.5–170.2][1]	0.729
Polymicrobial infection, n (%)	20 (22.2)	12 (20.3)	0.784
Staphylococcus aureus, n (%)	33 (36.7)	25 (42.4)	0.485
Coagulase-negative staphylococci (CoNS), n (%)	19 (21.1)	12 (20.3)	0.910
Positive blood cultures, n (%)	23 (25.6)	19 (32.2)	0.378

[1] Preoperative CRP was available in 90/90 patients with CONUT values and in 53/59 patients with missing CONUT components. CONUT: Controlling Nutritional Status; SD: standard deviation; IQR: interquartile range; JS-BACH: Joint-Specific BACH classification; CoNS: coagulase-negative staphylococci.

Supplementary Table S3. Expanded microbiology according to CONUT group

Variable	Overall cohort n= 90	High CONUT n=42	Low CONUT n=48	p value
Polymicrobial infection, n (%)	20 (22.2)	7 (16.7)	13 (27.1)	0.236
Staphylococcus aureus, n (%)	33 (36.7)	20 (47.6)	13 (27.1)	**0.044**
Methicillin-resistant Staphylococcus aureus (MRSA), n (%)	10 (11.1)	6 (14.3)	4 (8.3)	0.505
Coagulase-negative staphylococci (CoNS), n (%)	19 (21.1)	3 (7.1)	16 (33.3)	**0.002**
Difficult-to-treat organisms (DTT), n (%)	11 (12.2)	7 (16.7)	4 (8.3)	0.335
Gram-negative bacilli (GNB), n (%)	28 (31.1)	16 (38.1)	12 (25.0)	0.181
Culture-negative infection, n (%)	7 (7.8)	2 (4.8)	5 (10.4)	0.442
Positive blood cultures, n (%)	23 (25.6)	17 (40.5)	6 (12.5)	**0.002**

Bold indicates statistically significant differences ($p < 0.05$). Microbiological categories were not mutually exclusive. Difficult-to-treat organisms were defined using criteria adapted from Wimmer et al. (2020); fungi were included and Cutibacterium acnes was excluded, as it primarily represents diagnostic rather than therapeutic difficulty. CONUT: Controlling Nutritional Status; MRSA: methicillin-resistant Staphylococcus aureus; CoNS: coagulase-negative staphylococci; DTT: difficult-to-treat; GNB: Gram-negative bacilli.

Supplementary Table S4. MSIS-ORT distribution according to CONUT group. Data are presented as n (%)

MSIS-ORT category	Overall cohort n=90	High CONUT n=42	Low CONUT n=48
Tier 1. Infection control with no continued antibiotic therapy	42 (46.7)	9 (21.4)	33 (68.8)
Tier 2. Infection control with suppressive antibiotic therapy	0 (0.0)	0 (0.0)	0 (0.0)
Tier 3A. Aseptic revision at >1 year from initiation of PJI treatment	2 (2.2)	1 (2.4)	1 (2.1)
Tier 3B. Septic revision at >1 year from initiation of PJI treatment	2 (2.2)	2 (4.8)	0 (0.0)
Tier 3C. Aseptic revision at ≤1 year from initiation of PJI treatment	2 (2.2)	2 (4.8)	0 (0.0)
Tier 3D. Septic revision (including DAIR) at ≤1 year from initiation of PJI treatment	13 (14.4)	8 (19.0)	5 (10.4)
Tier 3E. Amputation, resection arthroplasty, or arthrodesis	3 (3.3)	3 (7.1)	0 (0.0)
Tier 3F. Retained spacer	0 (0.0)	0 (0.0)	0 (0.0)
Tier 4A. Death ≤1 year from initiation of PJI treatment	10 (11.1)	9 (21.4)	1 (2.1)
Tier 4B. Death >1 year from initiation of PJI treatment	16 (17.8)	8 (19.0)	8 (16.7)

Among the 2 tier 3B cases, the same pathogen as previously identified was isolated in 2/2 (100%). Among the 13 tier 3D cases, the same pathogen was isolated in 1/13 (7.69%), cultures were negative in 8/13

(61.54%), and superinfection occurred in 4/13 (30.77%) (2 methicillin-resistant *Staphylococcus epidermidis*; 1 methicillin-resistant *Staphylococcus aureus and* 1 *Proteus mirabilis*).

Supplementary Table S5. Exploratory biomarkers across outcomes. Values are presented as median [IQR] for patients with versus without each outcome.

Biomarker	Failure	p value	Total failure	p value	Overall mortality	p value
CRP, mg/L	113.0 [48.6–163.5] vs 44.3 [15.9–118.4]	0.051	63.3 [28.0–141.3] vs 46.4 [13.7–161.5]	0.532	52.6 [28.0–126.1] vs 48.6 [12.8–167.6]	0.815
CAR	37.2 [16.0–51.1] vs 14.0 [4.3–39.5]	**0.035**	23.7 [8.9–46.0] vs 14.4 [3.8–44.7]	0.321	15.1 [9.8–40.1] vs 15.7 [3.6–50.4]	0.558
NLR	4.8 [2.7–8.0] vs 3.6 [2.0–5.4]	0.099	4.5 [2.5–7.1] vs 3.5 [1.9–5.4]	0.095	3.9 [2.3–6.4] vs 3.4 [1.9–5.9]	0.230

Bold indicates statistically significant differences ($p<0.05$). CRP: C-reactive protein; CAR: CRP-to-albumin ratio; NLR: neutrophil-to-lymphocyte ratio; IQR: interquartile range.
